# Serum S100 calcium-binding protein A4 as a novel predictive marker of acute exacerbation of interstitial pneumonia after surgery for lung cancer

**DOI:** 10.1186/s12890-021-01554-y

**Published:** 2021-06-02

**Authors:** Atsushi Kagimoto, Yasuhiro Tsutani, Kei Kushitani, Takahiro Kambara, Takahiro Mimae, Yoshihiro Miyata, Yukio Takeshima, Morihito Okada

**Affiliations:** 1 Department of Surgical Oncology, Hiroshima University, 1-2-3, Kasumi, Hiroshima, 734-8551 Japan; 2 Department of Pathology, Hiroshima University, 1-2-3, Kasumi, Hiroshima, 734-8551 Japan

**Keywords:** Interstitial pneumonia, Acute exacerbation, S100A4, Lung cancer, Lung resection

## Abstract

**Background:**

Acute exacerbation (AE) of interstitial pneumonia (IP) is the most fatal complication after lung resection for lung cancer. To improve the prognosis of lung cancer with IP, the risk factors of AE of IP after lung resection should be assessed. S100 calcium-binding protein A4 (S100A4) is a member of the S100 family of proteins and is a known marker of tissue fibrosis. We examined the usefulness of S100A4 in predicting AE of IP after lung resection for lung cancer.

**Methods:**

This study included 162 patients with IP findings on preoperative high-resolution computed tomography scan who underwent curative-intent lung resection for primary lung cancer between April 2007 and March 2019. Serum samples were collected preoperatively. Resected lung tissue from 76 patients exhibited usual IP (UIP) pattern in resected lung were performed immunohistochemistry (IHC). Relationship between S100A4 and the incidence of AE of IP and short-term mortality was analyzed.

**Results:**

The receiver operating characteristic area under the curve for serum S100A4 to predict postoperative AE of IP was 0.871 (95% confidence interval [CI], 0.799–0.943; *P* < 0.001), with a sensitivity of 93.8% and a specificity of 75.3% at the cutoff value of 17.13 ng/mL. Multivariable analysis revealed that a high serum S100A4 level (> 17.13 ng/mL) was a significant risk factor for AE of IP (odds ratio, 42.28; 95% CI, 3.98–449.29; *P* = 0.002). A 1-year overall survival (OS) was significantly shorter in patients with high serum levels of S100A4 (75.3%) than in those with low serum levels (92.3%; *P* = 0.003). IHC staining revealed that fibroblasts, lymphocytes, and macrophages expressed S100A4 in the UIP area, and the stroma and fibrosis in the primary tumor expressed S100A4, whereas tumor cells did not.

**Conclusions:**

Serum S100A4 had a high predictive value for postoperative AE of IP and short-term mortality after lung resection.

**Supplementary Information:**

The online version contains supplementary material available at 10.1186/s12890-021-01554-y.

## Background

Interstitial pneumonia (IP), mostly idiopathic pulmonary fibrosis (IPF), is associated with an increasing risk of lung cancer [1, 2]. Approximately 4–6% of resected specimens of lung cancer showed some types of interstitial lung disease (ILD) [3]. The short-term mortality rate after lung resection for lung cancer has been improved; however, the major cause of death is acute exacerbation (AE) of IP, and the reported incidence and rate of mortality among patients with non-small cell lung cancer and AE with IP range from 0 to 32% and from 0 to 42%, respectively [4–6]. Among deaths of patients with IP and lung cancer, lung cancer is responsible for approximately 50%; the remaining deaths result from other causes, such as respiratory failure [3, 5]. To improve the prognosis of lung cancer with IP, the risk factors of AE of IP after lung resection should be assessed.

S100 calcium-binding protein A4 (S100A4) is a member of the S100 family of proteins and is a known marker of tissue fibrosis [7]. Reportedly, S100A4 promotes lung fibrosis via proliferation and activation of fibroblasts [8]. By inducing expression of α-smooth muscle actin (αSMA) and type 1 collagen, S100A4 also promotes the transition of fibroblasts to myofibroblasts [7]. Li et al. reported that a deficiency of S100A4 weakened pulmonary fibrosis; conversely, adoptive transfer of S100A4-positive macrophages induced lung injury/fibrosis in S100A4^−/−^ mice [9]. Based on these findings, S100A4 is assumed to play an important role in the pathogenesis of IPF. Recently, Akiyama et al. showed that high serum level of S100A4 was a significant predictive factor of IPF [10]. However, the significance of serum S100A4 level on the AE of IP after lung resection remained unknown. Therefore, we examined the relationship between S100A4 and AE of IP after lung resection for lung cancer.

## Methods

### Patients

This study was approved by the Ethics Committee of Hiroshima University Hospital (approval numbers Gen-38 and E-2098). Patient consent was obtained by using informed consent documents with an opt-out process. The study was carried out in accordance to institutional guidelines which is established based on the Declaration of Helsinki. Patients in whom IP had been diagnosed on preoperative high-resolution computed tomography (HRCT) and had undergone lung resection for primary lung cancer between April 2010 and March 2019 were included in this study. Twenty patients for whom preoperative serum samples were unavailable were excluded from this study. Among included patients, patients with UIP pattern in resected specimen was underwent immunohistochemistry (IHC). Flowchart of patient selection was shown in Additional file 1: Fig. S1. Chest HRCT, whole body [18F]-fluoro-2-deoxy-D-glucose positron emission tomography/CT, brain magnetic resonance imaging, and pulmonary function tests were conducted preoperatively to determine the indications for surgery, the appropriate surgical procedure, and clinical stage according to the eighth edition of the *TNM Classification for Lung Cancer* [11].

**Fig. 1:**
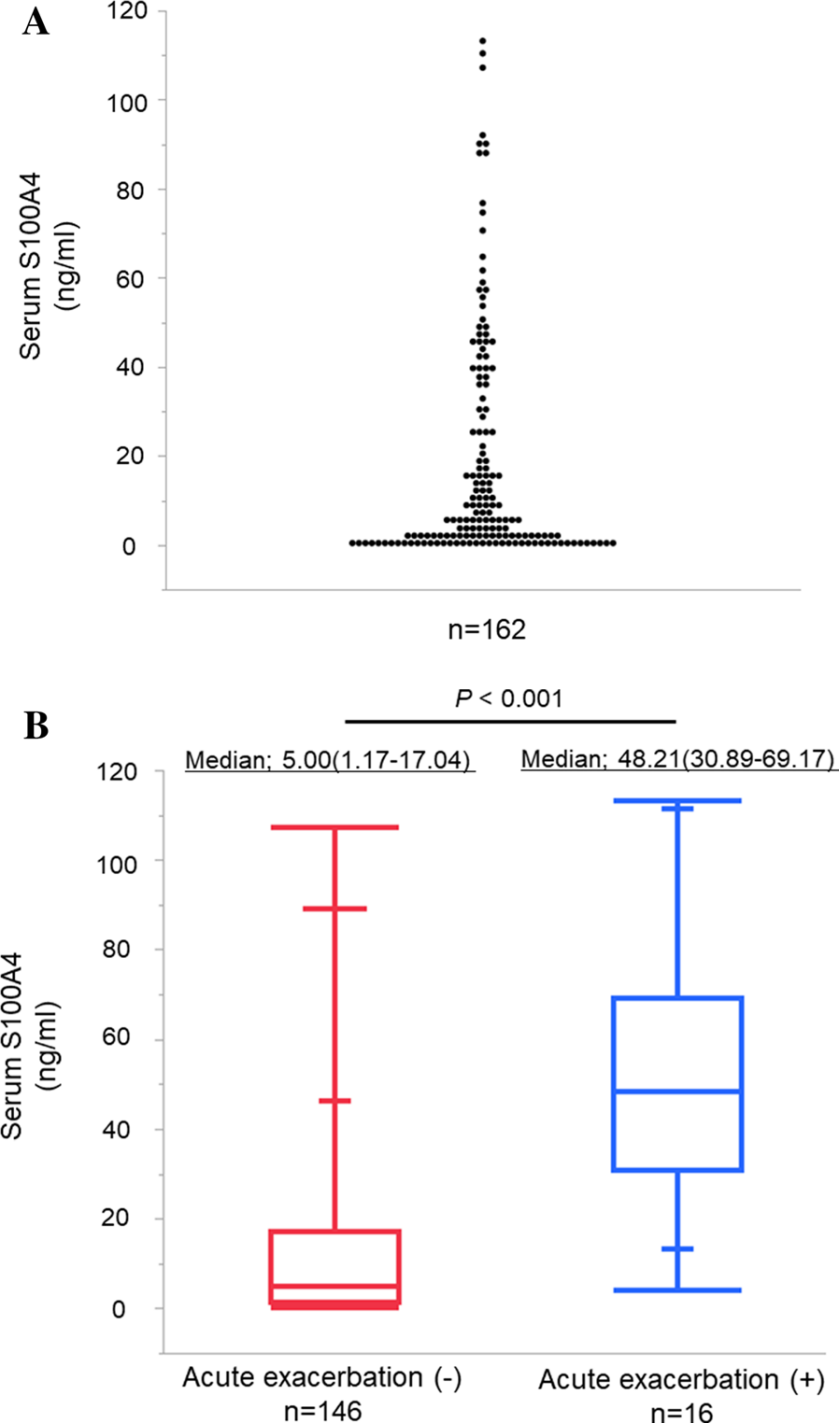
Serum levels of S100A4. **A** The median value of serum S100A4 was 5.87 ng/mL (interquartile range, 1.32–30.25 ng/mL). **B** Distribution of S100A4 levels according to presence or absence of AE of IP. The serum level of S100A4 was compared according to the presence or absence of AE of IP. S100A4 levels were significantly higher in patients with AE of IP (median 48.21 ng/mL; IQR 30.89–69.17 ng/mL) than in those without AE of IP (median 5.00 ng/mL; IQR 1.17–17.04 ng/mL; *P* < 0.001)

### HRCT

A 16-row multidetector CT was used to obtain chest images. We used the following parameters for high-resolution images of the lungs: 120 kVp, 200 mA, 1–2 mm section thickness, 512 × 512 pixel resolution, 0.5–1.0 s scanning time, a high-spatial reconstruction algorithm with a 20 cm field of view, and mediastinal (level, 40 HU; width, 400 HU) and lung (level, 600 HU; width, 1600 HU) window settings. ILD was defined radiologically according to the American Thoracic Society (ATS), European Respiratory Society (ERS), Japanese Respiratory Society (JRS), and Latin American Thoracic Association classifications (ALAT) and the patterns were classified as usual IP (UIP), possible UIP pattern, and inconsistent with UIP (Additional file 5: Table S1) [12]. Example images of each pattern are shown in Additional file 2: Fig. S2.

**Table 1:** Patient characteristics of all included patients and each S100A4 group

Variables	All patients n = 162	S100A4 high group n = 50	S100A4 low group n = 112	*P* value
Age, years (IQR)	74 (69–78)	72 (66–76)	74 (69–80)	0.136
Sex, male (%)	133 (82.1%)	44 (88.0%)	89 (79.5%)/	0.177
*Respiratory function*				
FVC (L) (IQR)	2.90 (2.34–3.39)	2.99 (2.44–3.56)	2.88 (2.30–3.36)	0.317
VC (L) (IQR)	2.93 (2.40–3.37)	3.03 (2.50–3.51)	2.89 (2.34–3.25)	0.330
%VC (%) (IQR)	92.2 (81.1–101.4)	92.4 (79.3–102.3)	92.2 (82.8–101.4)	0.701
%DLCO (%) (IQR)	54.9 (44.6–68.1)	57.7 (43.2–76.6)	53.7 (44.8–67.6)	0.360
Serum KL-6 (U / ml) (IQR)	443 (290–739)	473 (272–945)	437 (317–660)	0.065
Collagen disease (%)	12 (7.4%)	5 (10.0%)	7 (6.3%)	0.411
Preoperative steroid use (%)	11 (6.8%)	5 (10.0%)	6 (5.4%)	0.293
*Radiologic IP pattern*				0.836
UIP pattern (%)	54 (33.3%)	18 (36.0%)	36 (32.1%)	
Possible UIP pattern (%)	75 (46.3%)	23 (46.0%)	52 (46.4%)	
Inconsistent with UIP Pattern (%)	33 (20.4%)	9 (18.0%)	24 (21.4%)	
*Clinical stage*				0.144
0 (%)	4 (2.5%)	0 (0%)	4 (3.6%)	
I (%)	128 (79.0%)	42 (84.0%)	86 (76.8%)	
II (%)	21 (13.0%)	4 (8.0%)	17 (15.2%)	
III (%)	9 (5.6%)	4 (8.0%)	5 (4.5%)	
*Histology*				0.867
Adenocarcinoma (%)	65 (40.1%)	20 (40.0%)	45 (40.2%)	
Squamous cell Carcinoma (%)	62 (38.3%)	18 (36.0%)	44 (39.3%)	
Others (%)	35 (21.6%)	12 (24.0%)	23 (20.5%)	
*Surgical procedure*				0.494
Wedge resection (%)	48 (29.6%)	12 (24.0%)	36 (32.1%)	
Segmentectomy (%)	34 (21.0%)	13 (26.0%)	21 (18.8%)	
Lobectomy (%)	79 (48.8%)	25 (50.0%)	54 (48.2%)	
Pneumonectomy (%)	1 (0.6%)	0 (0%)	1 (0.9%)	
Operative time (min) (IQR)	148 (107–190)	160 (121–207)	139 (104–182)	0.069
*Pathological stage*				0.460
I	118 (72.8%)	35 (70.0%)	83 (74.1%)	
II	26 (16.0%)	8 (16.0%)	18 (16.1%)	
III	17 (10.5%)	6 (12.0%)	11 (9.8%)	
IV	1 (0.6%)	1 (2.0%)	0 (0%)	

*S100A4* S100 calcium-binding protein A4, *IQR* interquartile range, *FVC* forced vital capacity, *VC* vital capacity, *DLCO* diffusing capacity for carbon monoxide, *KL-6* krebs von den lungen-6, *IP* interstitial pneumonia, *UIP* usual interstitial pneumonia

**Fig. 2:**
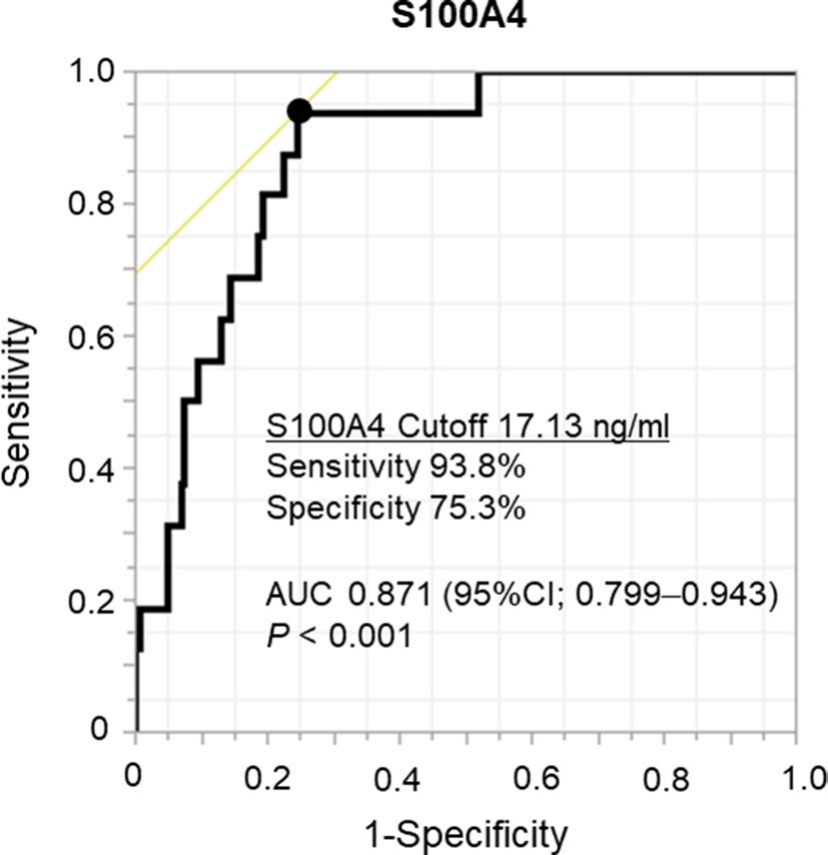
ROC curve of S100A4 predicting AE of IP. ROC curve analysis of the S100A4 level in predicting postoperative AE of IP (area under the curve 0.871; 95% CI 0.799–0.943; *P* < 0.001). The sensitivity and specificity of the cutoff S100A4 level (17.13 ng/mL) were 93.8% and 75.3%, respectively

### Serum S100A4 level measurement

Serum samples were obtained 1 day before surgery. The samples were stored at − 80 °C until measurement of S100A4. To measure S100A4, we used a commercially available enzyme-linked immunosorbent assay kit (CircuLex S100A4 ELISA Kit Ver.2; MBL Co., Ltd., Nagoya, Japan).

### Surgical procedure and evaluation of complications

Hybrid video-assisted thoracic surgery was conducted as an approach method [13]. Postoperative complications were evaluated according to the Clavien–Dindo classification [14]; complications of grade IIIa or worse were considered severe. The respiratory complications were AE of IP, bacterial pneumonia, bronchopleural fistula, pulmonary fistula that lasted more than 7 days or performed pleurodesis. Pleural effusion after chest tube removal was also included. AE of IP was defined by the following clinical characteristics within 30 days from surgery: (1) appearance or worsening of dyspnea, (2) deterioration of the interstitial shadow on CT, (3) decrease of SpO2 or PaO2 that was more severe than before surgery, and (4) no evidence of other cause of these findings [12].

### Pathological diagnosis and IHC

The pathological stage of lung cancer is based on the eighth edition of the *TNM Classification of Lung Cancer* [11]. Because the area of IP is not always resected due to the tumor location, among patients who measured serum S100A4, only patients who detected the UIP pattern in resected specimen were performed IHC. UIP pattern in resected specimen was diagnosed with following features according to the statement about IP from ATS/ERS/JRS/ALAT [12]: (1) evidence of marked fibrosis/architectural distortion and honeycombing in a predominantly subpleural/paraseptal distribution; (2) the presence of patchy involvement of lung parenchyma by fibrosis; (3) the presence of fibroblast foci; and (4) the absence of features that suggested an alternative diagnosis. The representative image of UIP pattern in resected specimen is shown in Additional file 3: Fig. S3.

**Fig. 3:**
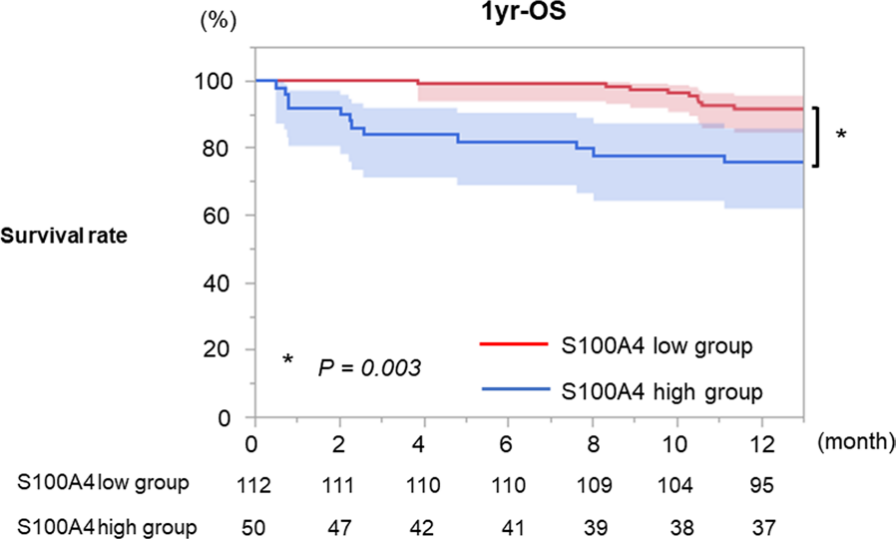
OS of each S100A4 group. The 1-year OS rate was significantly lower in patients with high S100A4 levels (75.3%; 95% CI 85.3–96.1) than in those with lower S100A4 levels (92.3%; 95% CI 85.3–96.1; *P* = 0.003)

To conduct the IHC study, we used 3 μm-thick tissue sections from representative formalin-fixed paraffin-embedded (FFPE) blocks of IP and the primary tumor and an immunohistochemical autostainer (BenchMark GX; Roche-Ventana Diagnostics, Tokyo, Japan) with the ultraView Universal DAB Detection Kit (Roche-Ventana Diagnostics). Rabbit anti-human S100A4 monoclonal antibody (Ab124805; Anti-S100A4 antibody; Abcam, Cambridge, UK), diluted 250 times with an antibody diluent (Roche-Ventana Diagnostics) were used as the primary antibody. Representative FFPE blocks of IP area and primary tumor were selected by pathologists (K.K and Y.T) who did not know the patients’ outcomes. The expression of S100A4 in IP area and primary tumor was evaluated.

### Statistical analysis

Data were presented as median and interquartile range (IQR) for continuous variables and n (%) for categorical variables. Categorical variables were compared using a chi-square test. Continuous variables were analyzed using the unpaired *t*-test. To evaluate the independent risk factors for AE of IP, a logistic regression model was used. Sex (male), presence of collagen disease, percent predicted vital capacity (%VC; ≤ 80%), percent diffusing capacity for carbon monoxide (%DLCO; ≤ 40%) [15], serum level of Krebs von den Lungen-6 (KL-6; ≥ 1000), preoperative steroid use, radiological pattern of IP (UIP pattern or not), operative time (continuous value), and anatomical resection (not wedge resection) were used in the multivariable analysis other than serum S100A4. The cutoff value of S100A4 was determined according to the receiver operating characteristic (ROC) curve predicting AE of IP. Overall survival (OS) was defined as the time interval from the date of surgery until the date of death from any cause or the date of the last follow-up visit. The 1-year OS rate after surgery was calculated by Kaplan–Meier method and compared by the log rank test. The JMP® software, Version 14 (SAS Institute, Cary, NC, USA) was used for all statistical analyses. A *P* value of < 0.05 was considered statistically significant for all analyses.

## Results

A total of 162 patients were included in this study and their characteristics are shown in Table 1. Twelve patients (7.4%) had ILD complicated by collagen disease and 11 patients (6.8%) were preoperative steroid users. Sixteen patients (9.9%) experienced AE of IP. The distribution of serum levels of S100A4 are shown in Fig. 1A. The median value of serum S100A4 was 5.87 ng/mL (IQR, 1.32–30.25 ng/mL). The serum values of S100A4 were compared according to the presence or absence of AE of IP (Fig. 1B). S100A4 level was significantly higher in patients with AE of IP (median, 48.21 ng/mL; IQR, 30.89–69.17 ng/mL) than in patients without AE of IP (5.00 ng/mL; IQR, 1.17–17.04 ng/mL; *P* < 0.001). There were no differences in the values of S100A4 between histological subtype of NSCLC (adenocarcinoma, 8.44 ng/mL [IQR, 1.01–30.29 ng/mL]; squamous cell carcinoma, 6.25 ng/mL [IQR, 1.39–33.70 ng/mL]; other subtypes, 6.26 ng/mL [IQR, 1.75–28.81 ng/mL; *P* = 0.814]. Based on the results of the ROC curve analysis of the S100A4 levels in predicting postoperative AE of IP (area under the curve [AUC], 0.871; 95% CI, 0.799–0.943; *P* < 0.001; Fig. 2), a cutoff value of 17.13 ng/mL was used to divide patients into two groups: 50 patients with high S100A4 levels (≥ 17.13 ng/mL) and 112 with low S100A4 levels (< 17.13 ng/mL). Patient characteristics of each group are also shown in Table 1. There were no significant differences between the characteristics of each group.

In the analysis of postoperative outcomes, AE of IP occurred in 14 patients (28.0%) with high S100A4 levels but in only 2 (1.8%) with low S100A4 levels (*P* < 0.001). The incidence of AE of IP more than grade IIIa was also higher among patients with high S100A4 levels (16.0%) than among those with low S100A4 levels (0%; *P* < 0.001). The incidence of any grade of respiratory complications was also higher among patients with high S100A4 levels (42.0%) than among those with low S100A4 levels (20.5%; *P* = 0.005). Among the patients with high S100A4 levels, the 30-day mortality rate was 8.0% (*P* = 0.002) and 90-day mortality rate was 16.0% (*P* < 0.001); both rates were 0% among the patients with low S100A4 levels (Table 2).

**Table 2:** Postoperative outcomes of each S100A4 group

	S100A4 high group (≥ 17.13 ng/ml) n = 50	S100A4 low group (< 17.13 ng/ml) n = 112	*P* value
AE of IP+ (any grade)	14 (28.0%)	2 (1.8%)	< 0.001
AE of IP+ (Grade IIIa)	8 (16.0%)	0 (0%)	< 0.001
Respiratory complications+ (any grade)	21 (42.0%)	23 (20.5%)	0.005
Respiratory complications+ (grade IIIa)	14 (28.0%)	17 (15.2%)	0.061
30-days mortality rate	4 (8.0%)	0 (0%)	0.002
90-days mortality rate	8 (16.0%)	0 (0%)	< 0.001
1-year OS rate	75.3%	92.3%	0.003

*S100A4* S100 calcium-binding protein A4, *AE* acute exacerbation, *IP* interstitial pneumonia, *OS* overall survival

Univariable and multivariable analyses were conducted to determine the risk factors for AE of IP. The ROC curve analysis of KL-6 level to predict AE of IP was conducted, but KL-6 level was not a significant predictive factor of AE of IP (AUC 0.671; 95% CI 0.520–0.821; *P* = 0.108; Additional file 4: Fig. S4). Therefore, the cutoff level of KL-6 was set at 1000 U/mL according to the past study [16]. In the univariable analysis, high S100A4 level, (odds ratio [OR] 21.39; 95% CI 4.67–98.64; *P* < 0.001), male (*P* = 0.010), low %VC (≤ 80%) (OR 4.21; 95%CI 1.46–12.20; *P* = 0.008), high KL-6 (≥ 1000) (OR 4.81; 95%CI 1.43–16.26; *P* = 0.011), and operative time (OR, 1.01; 95%CI 1.00–1.02; *P* = 0.048) were significant predictive factor of AE of IP. In the multivariable analysis, high S100A4 level (OR 42.28; 95%CI 3.98–449.29; *P* = 0.002), male (*P* = 0.033), low %VC (≤ 80%) (OR 10.20; 95%CI 1.26–82.26; *P* = 0.029), and operative time (OR 1.03; 95%CI 1.01–1.04; *P* = 0.001) were significant predictive factor of AE of IP (Table 3).

**Fig. 4:**
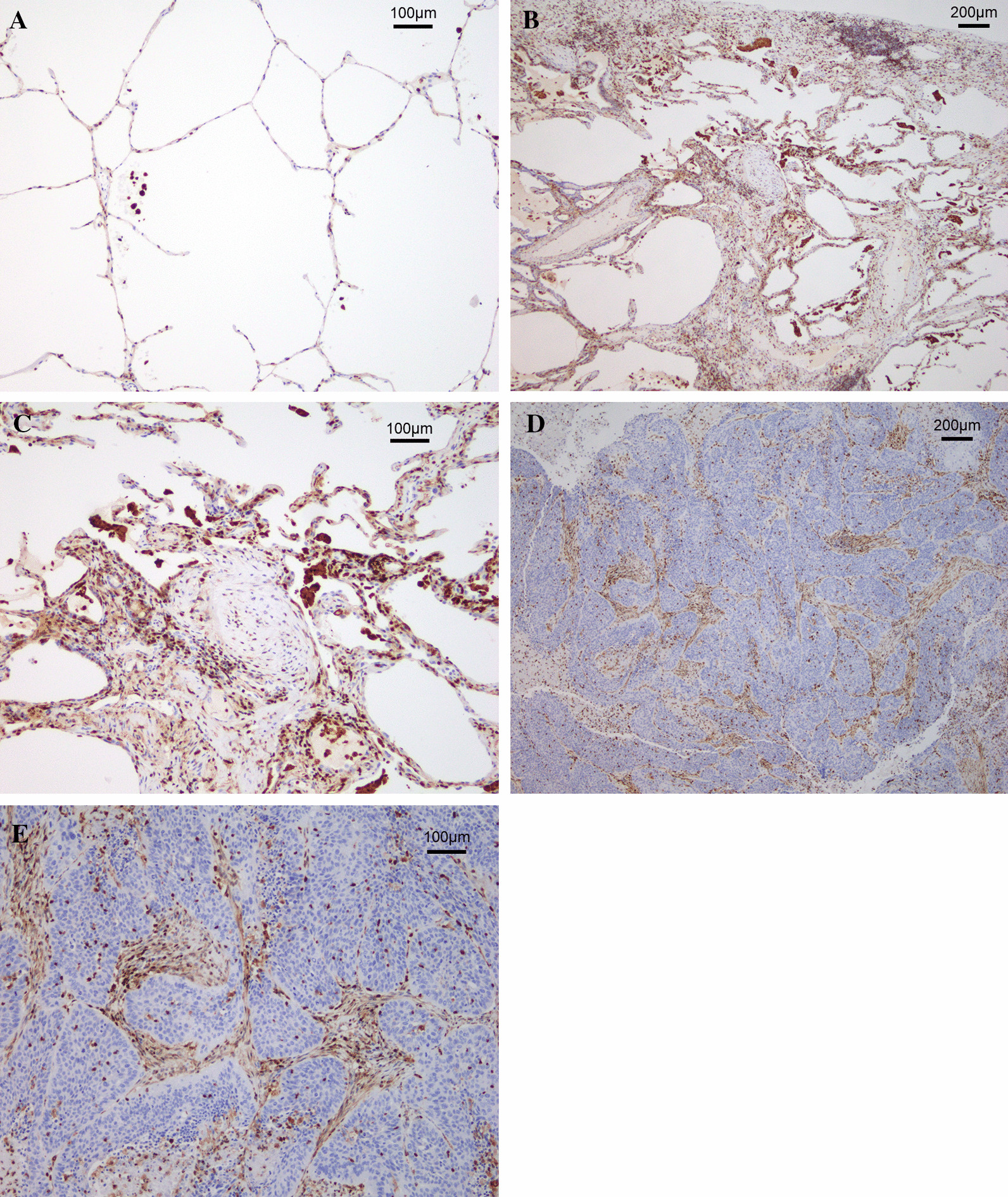
Representative images of S100A4 immunohistochemistry. **A** Representative image of normal lung tissue. In the normal areas of the lungs, S100A4 was sparsely expressed. **B**, **C** Representative images of areas of UIP with ×25 magnification (**B**) and ×100 magnification (**C**). The areas of UIP in all patients exhibited numerous S100A4-expressing cells such as fibroblasts, lymphocytes, and macrophages. **D**, **E** Representative images of the primary tumor with ×25 magnification (**D**) and ×100 magnification (**E**). In the primary tumor, S100A4 was expressed in the areas of stroma and fibrosis. Conversely, S100A4 was not expressed in the tumor cells

**Table 3:** Univariable and multivariable analysis of risk factor of acute exacerbation of interstitial pneumonia

	Univariable analysis		Multivariable analysis	
	OR (95%CI)	*P* value	OR (95%CI)	*P* value
S100A4 high (≥ 17.13 ng/ml)	21.39 (4.64–98.64)	< 0.001	42.28 (3.98–449.29)	0.002
Male	N/A*	0.010	N/A*	0.033
Collagen disease	0.82 (0.10–6.79)	0.852	0.01 (0.01–4.57)	0.141
%VC (≤ 80%)	4.21 (1.46–12.20)	0.008	10.20 (1.26–82.26)	0.029
DLCO (≤ 40%)	2.62 (0.83–8.31)	0.102	5.61 (0.30–103.97)	0.246
Preoperative steroid use	2.17 (0.43–11.08)	0.350	6.29 (0.22–180.26)	0.283
Radiologic UIP pattern	3.37 (1.13–10.02)	0.029	5.13 (0.624–42.22)	0.128
KL-6 (≥ 1000)	4.81 (1.43–16.26)	0.011	1.48 (0.09–24.59)	0.786
Operative time (continuous value)	1.01 (1.00–1.02)	0.048	1.03 (1.01–1.04)	0.001
Anatomical resection	1.27 (0.39–4.14)	0.694	3.21 (0.33–31.37)	0.315

*OR* odds ratio, *CI* confidence interval, *S100A4* S100 calcium-binding protein A4, *N/A* not available, *VC* vital capacity, *DLCO* diffusing capacity for carbon monoxide, *UIP* usual interstitial pneumonia, *KL-6* krebs von den lungen-6

*OR could not be calculated because none of female patients experienced AE of IP

The 1-year OS rate was significantly lower in the patients with high S100A4 levels (75.3%; 95% CI, 61.8–85.6) than in those with lower S100A4 levels (92.3%; 95% CI 85.3–96.1; *P* = 0.002; Table 2 and Fig. 3). There were no deaths due to lung cancer within 1-year from resection.

Among the resected samples, those from 76 patients exhibited a pathological UIP pattern and were subjected to IHC study. The characteristics of those 76 patients are shown in Additional file 6: Table S2. In the normal areas of the lungs, S100A4 was sparsely expressed in normal alveolar tissue (Fig. 4A). Conversely, the areas of UIP in all patients exhibited numerous S100A4-expressing cells such as fibroblasts, lymphocytes, and macrophages (Fig. 4B, C). In the primary tumor, S100A4 was expressed in the areas of stroma and fibrosis. Conversely, S100A4 was not expressed in the tumor cells (Fig. 4D, E).

## Discussion

The prognosis of lung cancer patients with IP is poorer than that of patients with normal lungs [17]. The complications of surgery can probably affect patients’ prognosis, and sublobar resection reduces the incidence of postoperative AE of IP and postoperative complications and reduces short-term mortality in patients with lung cancer with or without IP. Conversely, the incidence of pure solid tumors or squamous cell carcinomas, which are highly invasive characteristics, are higher among lung cancer patients with IP than among those without IP [3, 18]. Therefore, it is important to consider the balance between short-term and long-term outcome when considering the treatment strategy of lung cancer accompanied with IP. In this study, serum S100A4 level was a significant predictive factor for AE of IP. This will help clinicians decide on treatment strategy in lung cancer patients with IP.

S100A4 is known to play a key role in lung fibrosis. S100A4 is said to activate fibrogenic mesenchymal progenitor cells (MPCs) [19] that serve as cells of origin of disease-mediating myofibroblasts. Ex vivo analysis revealed that MPCs in IPF had increased levels of nuclear S100A4, which interacts with L-isoaspartyl methyltransferase to promote MPC self-renewal [20]. In vivo injection of human MPCs in IPF converted self-limited bleomycin-induced lung fibrosis in mice to persistent fibrosis in an S100A4-dependent manner. These results indicate that S100A4 confers fibrogenicity upon MPCs [20].

Extracellular S100A4 was also shown to activate lung fibroblasts [19], and the main mechanism of activation is assumed to be the upregulation of αSMA and type I collagen through the increase of sphingosine-1-phosphate [7, 21]. Macrophages have been suggested as the origin of extracellular S100A4 [9], which is consistent with the large number of S100A4-positive macrophages that we found in the areas of UIP, and it is reasonable that the serum level of S100A4 can reflect IP activity. Several studies have shown that expression of S100A4 in IHC study is a prognostic factor for lung cancer [22, 23], and lung cancer subtype or stage can affect the serum level of S100A4. In our study, however, patients with high serum levels of S100A4 had almost the same subtypes and stages as did patients with low serum levels of S100A4. Additionally, in our study, S100A4 was sparsely expressed in normal alveolar tissue and in lung tissues with IPF in all patients who exhibited numerous S100A4-expressing cells. As for the main tumor, in the majority of patients, S100A4 was expressed only in the areas of stroma and fibrosis but not in the tumor cell. From these findings, we speculate that main origin of serum S100A4 is lung fibrosis such as IP areas and there is little bias due to lung cancers. Furthermore, S100A4 is secreted into the extracellular matrix, and this activates inflammatory signals [21]. Therefore, results of this study that serum S100A4 is a useful predictive factor for AE of IP are making sense.

Several researchers have examined whether S100A4 could be a therapeutic target. Niclosamide, an approved antihelminthic drug for the treatment of tapeworm infections, inhibited metastasis formation in a mouse model of colon cancer by blocking S100A4 expression and its effects on the WNT/CTNNB1 signaling pathway [24]. In a mouse model of IP, niclosamide inhibited the expression of S100A4 [8]. Paclitaxel has also been reported to inhibit invasion and hematogenous metastasis of cholangiocarcinoma by downregulating nuclear S100A4 [25]. Therefore, S100A4 can be not only a predictive factor of AE of IP but also a therapeutic target in patients with IP and lung cancer in the future. In our study, we measured only serum S100A4 and IHC of resected lung specimens; therefore, the exact origin of S100A4 remains unknown, and it may be necessary to use cell lines or animal models to examine whether it is a significant therapeutic target.

This study had several limitations. First, at present, S100A4 cannot be easily measured in daily clinical practice. Second, this study was retrospective and conducted at a single institute. The number of included patients was small—although, being from a single institution, the cohort was relatively large—and in multivariable analysis, the well-known risk factors of AE of IP, such as DLCO or UIP pattern on preoperative CT, were not significant predictive factors. Therefore, confirmation by a large trial, such as Japan Clinical Oncology Group 1708 [26], is necessary. Third, although only the specimens with UIP pattern were subjected to IHC study, there may be some bias in histological examinations of this study because the areas of the most severe IP are not always resected. However, our findings will contribute to the process of deciding on treatment strategy of lung cancer with IP.

## Conclusions

Among patients with high serum levels of S100A4, AE of IP occurred more frequently and the short-term mortality rate after surgery was higher than those with low serum levels of S100A4. Although a study with a larger sample size is necessary to confirm our findings, the serum level of S100A4 appears to be a significant predictor of AE of IP after lung resection for lung cancer.

## Supplementary Information

**Additional file 1: Fig. S1**. Flowchart of patient selection. A total of 182 patients underwent curative-intent resection for primary lung cancer. Serum samples were obtained before resection in 162 patients, and the S100A4 level was measured. UIP was diagnosed in surgically resected specimens from 76 patients, and these specimens were subjected to immunohistochemistry for S100A4.

**Additional file 2: Fig. S2**. Representative computed tomographic images of IP. Representative images of UIP pattern (A), possible UIP pattern (B), and pattern inconsistent with UIP (C) according to the ATS, ERS, JRS, and ALTA classifications.

**Additional file 3: Fig. S3**. Representative image of UIP pattern. UIP pattern in resected specimens was diagnosed based on the following features, according to the guidelines of IP from the ATS, ERS, JRS, and ALTA: (1) evidence of marked fibrosis/architectural distortion and honeycombing in a predominantly subpleural/paraseptal distribution; (2) presence of patchy involvement of lung parenchyma by fibrosis; (3) presence of fibroblast foci (*); and (4) absence of features suggesting an alternative diagnosis.

**Additional file 4: Fig. S4**. ROC curve of KL-6 level to predict AE of IP. ROC curve analysis of the level of KL-6 in predicting postoperative AE of IP (area under the curve, 0.671; 95% confidence interval, 0.520–0.821; *P* = 0.108).

**Additional file 5: Table S1**. Computed tomography findings of interstitial pneumonia.

**Additional file 6: Table S2**. Characteristics of patients who underwent immunohistochemistry.

## Data Availability

All data generated or analyzed during this study are included in this published article.

## Abbreviations

AE: Acute exacerbation
ALTA: Latin American Thoracic Association classifications
SMA: α-Smooth muscle actin
ATS: American Thoracic Society
AUC: Area under the curve
CI: Confidence interval
CT: Computed tomography
ELISA: Enzyme-linked immune sorbent assay
ERS: European Respiratory Society
FFPE: Formalin-fixed paraffin-embedded
FVC: Forced vital capacity
HRCT: High-resolution computed tomography
IHC: Immunohistochemistry
ILD: Interstitial lung disease
IP: Interstitial pneumonia
IPF: Idiopathic pulmonary fibrosis
IQR: Interquartile range
JRS: Japanese Respiratory Society
KL-6: Krebs von den lungen-6
OR: Odds ratio
OS: Overall survival
ROC: Receiver operating characteristic
S100A4: S100 calcium-binding protein A4
UIP: Usual interstitial pneumonia
%DLCO: Percent diffusing capacity for carbon monoxide
%VC: Percent predicted vital capacity

## References

[1] Hubbard R, Venn A, Lewis S, Britton J (2000). Lung cancer and cryptogenic fibrosing alveolitis. A population-based cohort study. Am J Respir Crit Care Med.

[2] Park J, Kim DS, Shim TS (2001). Lung cancer in patients with idiopathic pulmonary fibrosis. Eur Respir J.

[3] Sato T, Watanabe A, Kondo H (2015). Long-term results and predictors of survival after surgical resection of patients with lung cancer and interstitial lung disease. J Thorac Cardiovasc Surg.

[4] Amano J, Kuwano H, Yokomise H (2013). Thoracic and cardiovascular surgery in Japan during 2011. Annual report by the Japanese Association for Thoracic Surgery. Gen Thorac Cardiovasc Surg.

[5] Watanabe A, Miyajima M, Mishina T (2013). Surgical treatment for primary lung cancer combined with idiopathic pulmonary fibrosis. Gen Thorac Cardiovasc Surg.

[6] Sato T, Teramukai S, Kondo H (2014). Impact and predictors of acute exacerbation of interstitial lung diseases after pulmonary resection for lung cancer. J Thorac Cadiovasc Surg.

[7] Fei F, Qu J, Li C, Wang X, Li Y, Zhang S (2017). Role of metastasis-induced protein S100A4 in human non-tumor pathophysiologies. Cell Biosci.

[8] Zhang W, Ohno S, Steer B (2018). S100a4 is secreted by alternatively activated alveolar macrophages and promotes activation of lung fibroblasts in pulmonary fibrosis. Front Immunol.

[9] Li Y, Bao J, Bian Y (2018). S100A4+ macrophages are necessary for pulmonary fibrosis by activating lung fibroblasts. Front Immunol.

[10] Akiyama N, Hozumi H, Isayama T (2020). Clinical significance of serum S100 calcium-binding protein A4 in idiopathic pulmonary fibrosis. Respirology.

[11] Goldstraw P, Chansky K, Crowley J (2016). The IASLC lung cancer staging project: proposals for revision of the TNM stage groupings in the forthcoming (Eighth) edition of the TNM classification for lung cancer. J Thorac Oncol.

[12] Raghu G, Collard HR, Egan JJ (2011). An official ATS/ERS/JRS/ALAT statement: idiopathic pulmonary fibrosis: evidence-based guidelines for diagnosis and management. Am J Respir Crit Care Med.

[13] Okada M, Sakamoto T, Yuki T, Mimura T, Miyoshi K, Tsubota N (2005). Hybrid surgical approach of video-assisted minithoracotomy for lung cancer: significance of direct visualization on quality of surgery. Chest.

[14] Clavien PA, Barkun J, de Oliveira ML (2009). The Clavien-Dindo classification of surgical complications; five-year experience. Ann Surg.

[15] Ohsawa M, Tsutani Y, Fujiwara M, Mimae T, Miyata Y, Okada M (2020). Predicting severe postoperative complication in patients with lung cancer and interstitial pneumonia. Ann Thorac Surg.

[16] Sato T, Kondo H, Watanabe A (2015). A simple risk scoring system for predicting acute exacerbation of interstitial pneumonia after pulmonary resection in lung cancer patients. Gen Thorac Cardiovasc Surg.

[17] Tsutani Y, Mimura T, Kai Y (2017). Outcomes after lobar versus sublobar resection for clinical stage I non-small cell lung cancer in patients with interstitial lung disease. J Thorac Cardiovasc Surg.

[18] Oh SY, Kim MY, Kim JE (2015). Evolving early lung cancers detected during follow-up of idiopathic interstitial pneumonia: serial CT features. Am J Roentgenol.

[19] Xia H, Bodempudi V, Benyumov A (2014). Identification of a cell-of-origin for fibroblasts comprising the fibrotic reticulum in idiopathic pulmonary fibrosis. Am J Pathol.

[20] Xia H, Gilbertsen A, Herrera J (2017). Calcium-binding protein S100A4 confers mesenchymal progenitor cell fibrogenicity in idiopathic pulmonary fibrosis. J Clin Invest.

[21] Li Z, Li Y, Liu S, Qin Z (2020). Extracellular S100A4 as a key player in fibrotic diseases. J Cell Mol Med.

[22] Zhang H, Liu J, Yue D (2013). Clinical significance of E-cadherin, β-catenin, vimentin and S100A4 expression in completely resected squamous cell lung carcinoma. J Clin Pathol.

[23] Miyazaki N, Abe Y, Oida Y (2006). Poor outcome of patients with pulmonary adenocarcinoma showing decreased E-cadherin combined with increased S100A4 expression. Int J Oncol.

[24] Sack U, Walther W, Scudiero D (2011). Novel effect of antihelminthic niclosamide on S100A4-mediated metastatic progression in colon cancer. J Natl Cancer Inst.

[25] Cadamuro M, Spagnuolo G, Sambado L (2016). Low-dose paclitaxel reduces S100A4 nuclear import to inhibit invasion and hematogenous metastasis of cholangiocarcinoma. Cancer Res.

[26] Tanaka K, Tsutani Y, Wakabayashi M (2020). Sublobar resection versus lobectomy for patients with resectable stage I non-small cell lung cancer with idiopathic pulmonary fibrosis: a phase III study evaluating survival (JCOG1708, SURPRISE). Jpn J Clin Oncol.

