# Supplementary figures and images for: Serum S100 calcium-binding protein A4 as a novel predictive marker of acute exacerbation of interstitial pneumonia after surgery for lung cancer
Source: BMC Pulm Med. 2021 Jun 2;21:186. doi: 10.1186/s12890-021-01554-y (PMC8173829; doi:10.1186/s12890-021-01554-y)

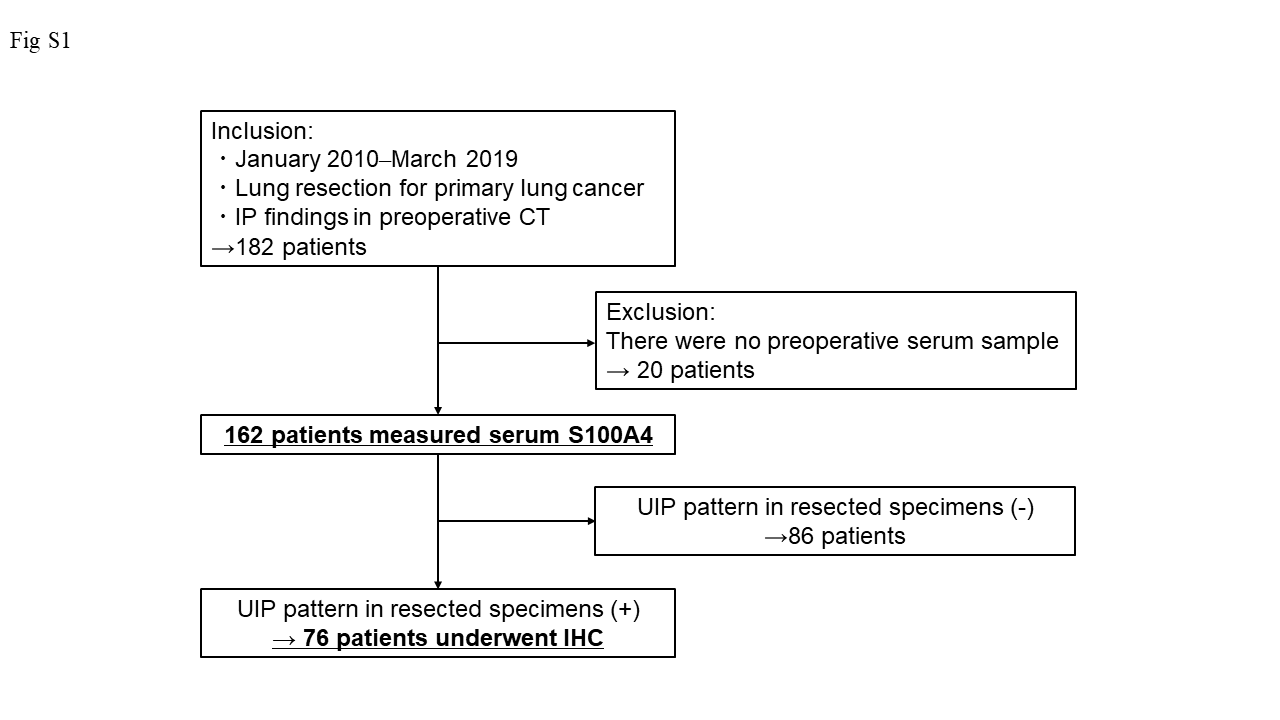

Supplement: Supplementary file 1 — Additional file 1: Fig. S1. Flowchart of patient selection. A total of 182 patients underwent curative-intent resection for primary lung cancer. Serum samples were obtained before resection in 162 patients, and the S100A4 level was measured. UIP was diagnosed in surgically resected specimens from 76 patients, and these specimens were subjected to immunohistochemistry for S100A4. [file 12890_2021_1554_MOESM1_ESM.tif]

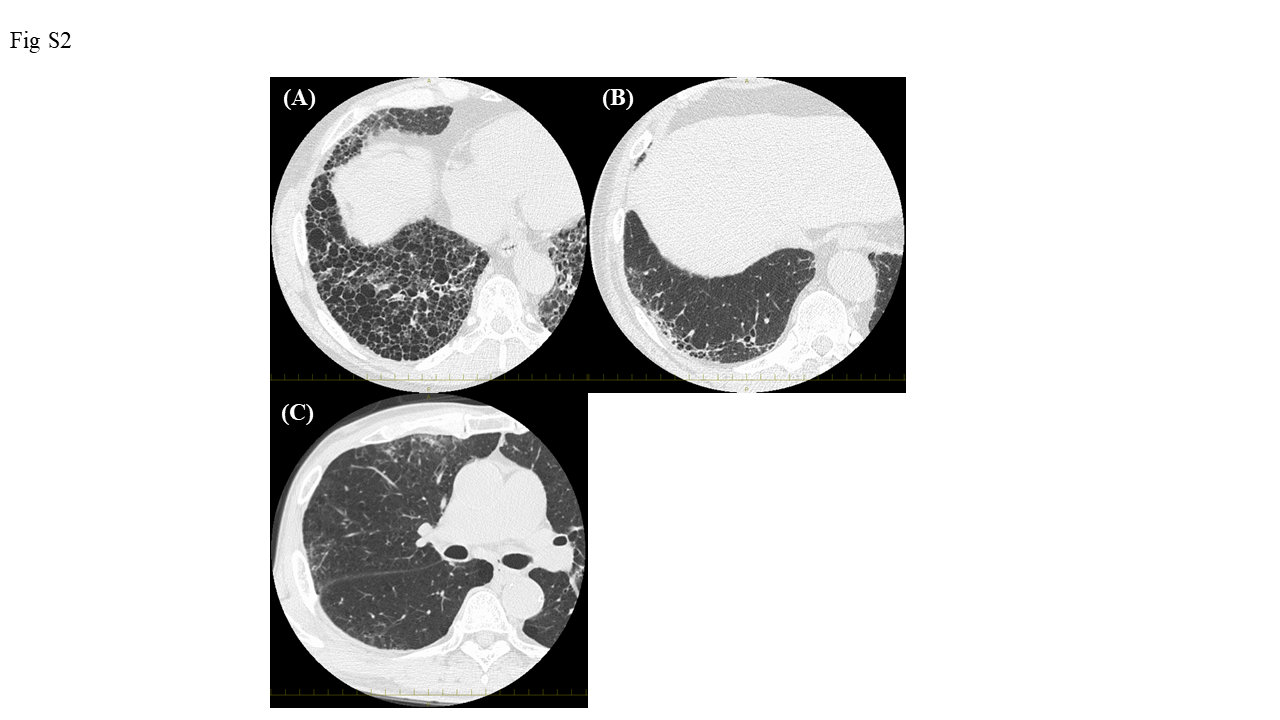

Supplement: Supplementary file 2 — Additional file 2: Fig. S2. Representative computed tomographic images of IP. Representative images of UIP pattern (A), possible UIP pattern (B), and pattern inconsistent with UIP (C) according to the ATS, ERS, JRS, and ALTA classifications. [file 12890_2021_1554_MOESM2_ESM.tif]

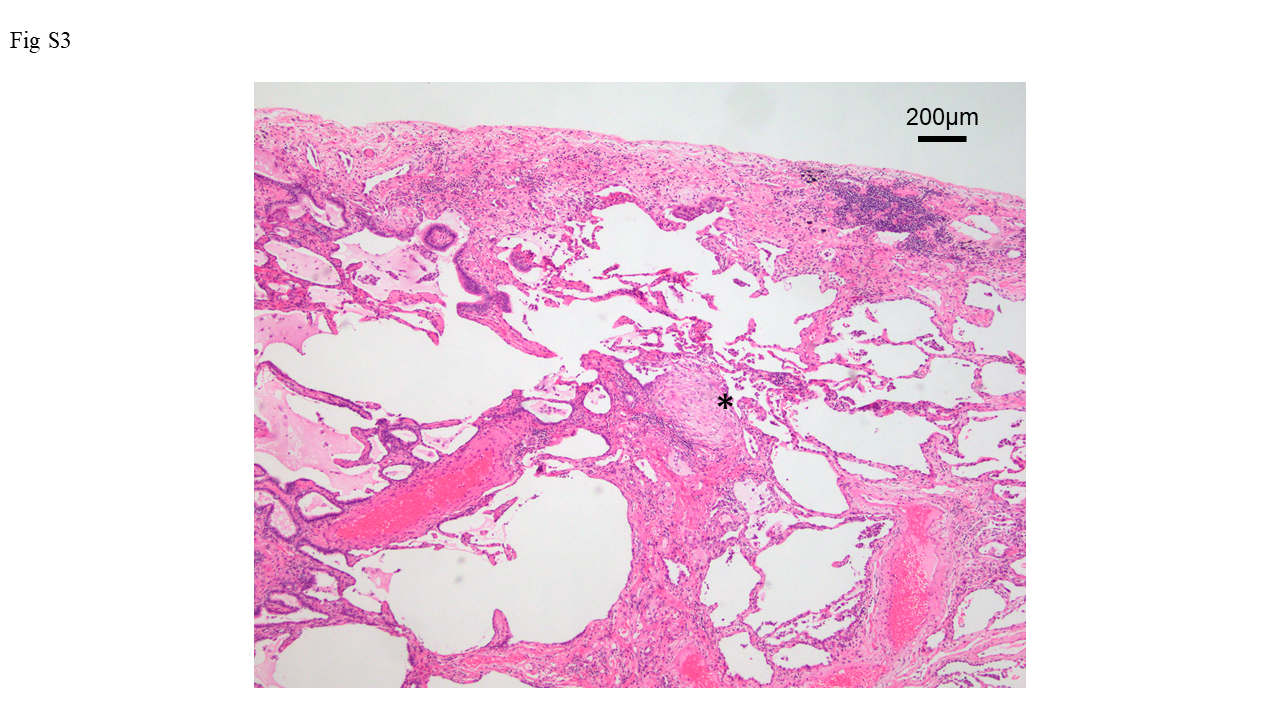

Supplement: Supplementary file 3 — Additional file 3: Fig. S3. Representative image of UIP pattern. UIP pattern in resected specimens was diagnosed based on the following features, according to the guidelines of IP from the ATS, ERS, JRS, and ALTA: (1) evidence of marked fibrosis/architectural distortion and honeycombing in a predominantly subpleural/paraseptal distribution; (2) presence of patchy involvement of lung parenchyma by fibrosis; (3) presence of fibroblast foci (*); and (4) absence of features suggesting an alternative diagnosis. [file 12890_2021_1554_MOESM3_ESM.tif]

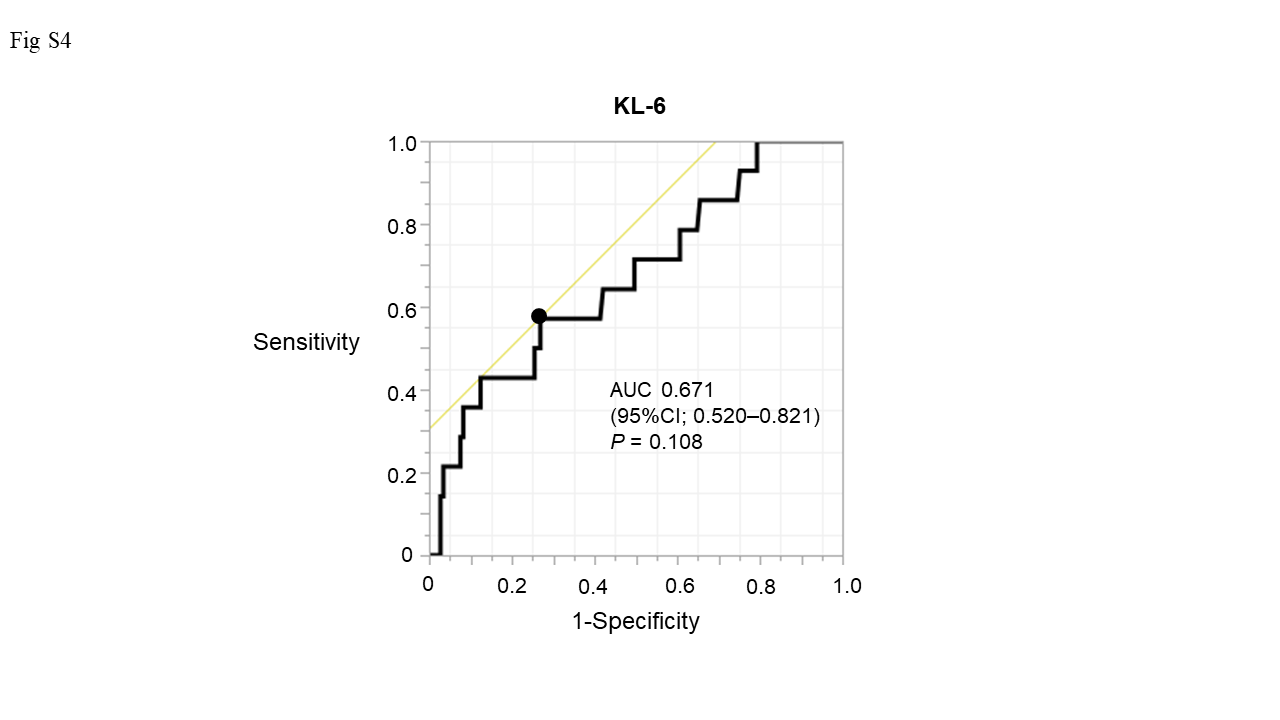

Supplement: Supplementary file 4 — Additional file 4: Fig. S4. ROC curve of KL-6 level to predict AE of IP. ROC curve analysis of the level of KL-6 in predicting postoperative AE of IP (area under the curve, 0.671; 95% confidence interval, 0.520–0.821; P = 0.108). [file 12890_2021_1554_MOESM4_ESM.tif]
