# Supplementary material for: Serum S100 calcium-binding protein A4 as a novel predictive marker of acute exacerbation of interstitial pneumonia after surgery for lung cancer
Source: BMC Pulm Med. 2021 Jun 2;21:186. doi: 10.1186/s12890-021-01554-y (PMC8173829; doi:10.1186/s12890-021-01554-y)
Supplement: Supplementary file 5 — Additional file 5: Table S1. Computed tomography findings of interstitial pneumonia. [file 12890_2021_1554_MOESM5_ESM.docx]

**Supplemental Table 1. Computed tomography findings of interstitial pneumonia**

| IP pattern | Findings |
| --- | --- |
| UIP pattern  (all four features) | subpleural, basal predominance; reticular abnormality; honeycombing with or without traction bronchiectasis; and absence of features listed as inconsistent with UIP pattern |
| Possible UIP pattern  (all three features) | subpleural, basal predominance; reticular abnormality; and absence of features listed as inconsistent with UIP pattern |
| Inconsistent with UIP pattern (any of the seven features) | upper or mid-lung predominance, peribronchovascular predominance, extensive ground-glass abnormality (extent > reticular abnormality), profuse micronodules (bilateral predominantly upper lobes), discrete cysts (multiple, bilateral, and away from areas of honeycombing), diffuse mosaic attenuation/air trapping (bilateral, in three or more lobes), or consolidation in bronchopulmonary segment(s)/lobe(s) |

UIP, usual interstitial pneumonia.
