# Supplementary material for: Serum S100 calcium-binding protein A4 as a novel predictive marker of acute exacerbation of interstitial pneumonia after surgery for lung cancer
Source: BMC Pulm Med. 2021 Jun 2;21:186. doi: 10.1186/s12890-021-01554-y (PMC8173829; doi:10.1186/s12890-021-01554-y)
Supplement: Supplementary file 6 — Additional file 6: Table S2. Characteristics of patients who underwent immunohistochemistry. [file 12890_2021_1554_MOESM6_ESM.docx]

**Supplemental Table 2. Characteristics of patients who underwent immunohistochemistry**

| Variables | n = 76 |
| --- | --- |
| Age, years (IQR) | 74 (69–78) |
| Sex, male (%) | 63 (82.9%) |
| Respiratory function |  |
| FVC (L) (IQR) | 2.94 (2.26–3.33) |
| VC (L) (IQR) | 3.02 (2.26–3.32) |
| %VC (%) (IQR) | 92.0 (75.8–101.4) |
| %DLCO (%) (IQR) | 49.0 (40.1–63.0) |
| serum KL-6 (U / ml) (IQR) | 576 (388–932) |
| Collagen disease | 3 (4.0%) |
| Preoperative steroid use | 6 (7.7%) |
| Radiologic IP pattern |  |
| UIP pattern (%) | 43 (56.6%) |
| Possible UIP pattern (%) | 29 (38.3%) |
| Inconsistent with UIP pattern (%) | 4 (5.3%) |
| Clinical stage |  |
| 0 (%) | 1 (1.3%) |
| I (%) | 59 (77.6%) |
| II (%) | 9 (11.8%) |
| III (%) | 7 (9.2%) |
| Histology |  |
| Adenocarcinoma (%) | 20 (26.3%) |
| Squamous cell carcinoma (%) | 36 (47.4%) |
| Others (%) | 20 (26.3%) |
| Surgical procedure |  |
| Wedge resection (%) | 36 (47.4%) |
| Segmentectomy (%) | 11 (14.5%) |
| Lobectomy (%) | 29 (38.2%) |
| Operative time (min) (IQR) | 141 (94–165) |
| Pathological stage |  |
| I | 53 (69.7%) |
| II | 11 (14.5%) |
| III | 12 (15.8%) |
| Serum S100A4 | 5.51 (1.13–29.20) |

IQR, interquartile range; FVC, forced vital capacity; VC, vital capacity; DLCO, diffusing capacity for carbon monoxide; KL-6, krebs von den lungen-6; IP, interstitial pneumonia; UIP, usual interstitial pneumonia; S100A4, S100 calcium-binding protein A4;
